# Carcinoid tumorlet in pulmonary sequestration with bronchiectasis after breast cancer: A case report

**DOI:** 10.3892/ol.2013.1210

**Published:** 2013-02-22

**Authors:** YIWANG YE, ZHIMIN MU, DA WU, YUANCAI XIE

**Affiliations:** Department of Thoracic Surgery, Peking University Shenzhen Hospital, Shenzhen, Guangdong 518036, P.R. China

**Keywords:** pulmonary sequestration, carcinoid tumorlet, bronchiectasis, breast cancer

## Abstract

Pulmonary sequestration (PS) is an uncommon lung disease. Carcinoid tumorlets in pulmonary sequestration are extremely rare. This case report presents a rare clinical case of carcinoid tumorlet in pulmonary sequestration with bronchiectasis after breast cancer. A 64-year-old female was diagnosed with infiltrating ductal carcinoma of the left breast in February 2009. Chest computer tomography (CT) revealed a cystic low-density mass of ∼2.5×4.7 cm in the right lower lung field, as well as cystic bronchiectasis in the right lower lobe. A right lower lobectomy was performed. In the surgery, abnormal vessel growth from the mass was found. Therefore, intralobar PS was diagnosed and pathological examination supported the diagnosis. Subsequently, pathological examination identified a carcinoid tumorlet in the PS. This report presents a rare clinical case of PS and bronchiectasis as well as carcinoid tumorlet in PS following diagnosis of breast cancer three years earlier. When a mass is found in the lung of patents with bronchiectasis with a history of breast cancer, aggressive therapy should be considered, since the mass may be a tumor or precancerous lesion.

## Introduction

Pulmonary sequestration (PS) is an uncommon congenital disease defined as a segment of lung parenchyma separated from the tracheobronchial tree and receiving its blood supply from a systemic artery rather than a pulmonary arterial branch (1).

Pulmonary carcinoid tumorlet is a nodular proliferation of neuroendocrine cells which is not larger than 5 mm (2). It is rarely observed in conjunction with pulmonary sequestration and pulmonary neuroendocrine tumorlets (3). This report presents a rare clinical case of carcinoid tumorlet in pulmonary sequestration with bronchiectasis following breast cancer. This study was approved by the ethics committee of Peking University Shenzhen Hospital, China. Written informed consent was obtained from the patient.

## Case report

**Figure 1:**
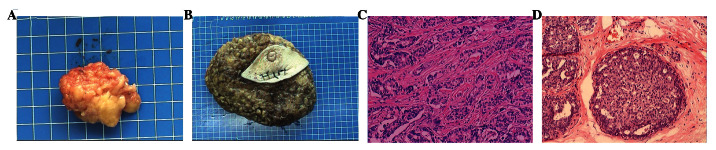
Pathological examination of resected breast cancer. (A) The macroscopic section of the resected breast mass. (B) The macroscopic section of the resected left breast. The pathological specimens demonstrates (C) poorly differentiated infiltrating ductal carcinoma and (D) the metastatic lymph node (H&E staining, ×100).

A 64-year-old female first presented to the Department of Thyroid and Breast Surgery, Peking University Shenzhen Hospital with a palpable mass in her left breast in February 2009. The color ultrasound and mammary gland molybdenum revealed breast cancer. The patient underwent lump resection and rapid pathological examination revealed breast cancer, therefore she subsequently underwent mastectomy with axillary lymph node dissection. The pathological diagnosis was infiltrating ductal carcinoma of the left breast (pT1, N1, M0, estrogen receptor-positive, progesterone receptor-positive) in February 2009 (Fig. 1). The patient received four cycles of adjuvant chemotherapy with epirubicin and cyclophosphamide, followed by four cycles of docetaxel. She completed treatment in August 2009. She underwent regular reexamination every 6 months, and there were no signs of recurrence or metastasis for several years.

**Figure 2:**
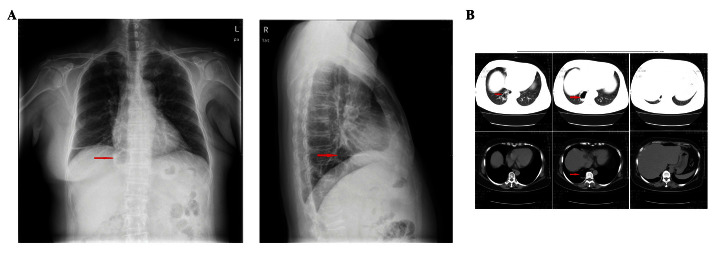
Images before lower lobectomy surgery. (A) The chest X-ray showed a striped high-density mass in the right lung above the diaphragmatic surface (arrow). (B) Chest computer tomography (CT) revealed a cystic low-density mass of ∼2.5×4.7 cm in the right lower lung field (arrow), as well as cystic bronchiectasis and pneumonia in the right lower lobe.

In July 2012, a chest X-ray showed a striped high-density mass in the right lung above the diaphragmatic surface upon routine reexamination. Chest computer tomography (CT) revealed a cystic low-density mass of ∼2.5×4.7 cm in the right lower lung field, as well as cystic bronchiectasis in the right lower lobe (Fig. 2). The patient was diagnosed with a pulmonary cyst with infection and bronchiectasis in the right lower lobe and was treated with antibiotics. After the infection was controlled, a right lower lobectomy was performed. In the surgery, systemic arterial supply from the descending thoracic aorta was observed, as well as venous drainage to the left inferior pulmonary vein. Therefore, intralobar pulmonary sequestration was diagnosed. The pathologists supported this diagnosis.

**Figure 3:**
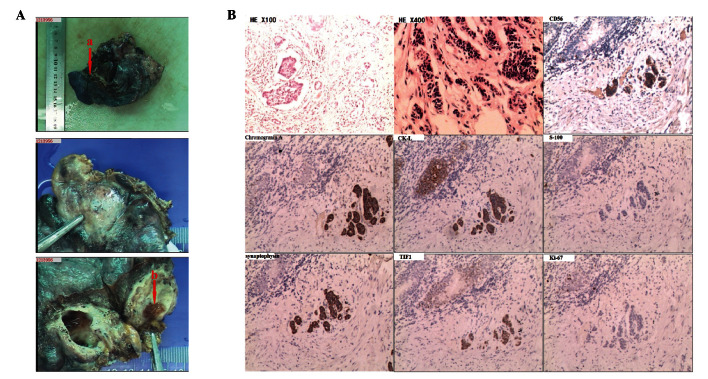
(A) Histological examination of resected lung tissue (H&E staining, ×100/×400). The arrow (a) shows the abnormal artery from the descending thoracic aorta to the resected mass. The arrow (b) shows a nodule in the mass. (B) Using immunohistochemistry, the carcinoma cells were positive for CD56, CK-L, synaptophysin, TIF1, cromogranin A and Ki-67 (<1% positive) and negative for S-100 (magnification, ×100).

The resected mass measured 5×4×1 cm and showed cystic space interposed by bronchial sample ciliated columnar epithelium and cartilage structures. A nodule was identified in the cystic wall, spanning 0.4 cm. The cells were arranged in nests and cords without stromal reaction, and showed salt-and-pepper type nuclear features without prominent nucleoli (Fig. 3). The immnohistochemical study revealed that the tumor cells were positive for synaptophysin, chromogranin A, CK-L, Ki-67 (<1% positive), TIF1 and CD56, and negative for S-100 (Fig. 4). The nest and cord-like growth pattern and cellular features suggested a carcinoid tumorlet.

## Discussion

PS is deemed a rare congenital malformation. It comprises 0.15–6.4% of all congenital pulmonary anomalies and is more common in the left lung and lower lobes (4). Anatomically, it has been classically described in two different forms: intralobar and extralobar. With intralobar pulmonary sequestration, which comprises 75% of all PS, the abnormal tissue is partly surrounded by normal lung, so it shares the same pleura as normal lung tissue, while the extralobar form is separated from normal lung tissue with its own pleura and maintains complete anatomical and physiological separation between the cyst and the adjacent normal lung (5). In the case presented here, the lung parenchyma was found to share the same pleura with normal lung tissue, and is therefore the intralobar type.

It has also been claimed that intralobar sequestration may arise as an acquired lesion, possibly secondary to local infection, such as bronchiectasis (6,7). In the present case, the chest CT showed bronchiectasis in right lower lung field and a cystic low-density mass, which was confirmed to be PS following surgery. It was therefore assumed that the PS was connected with bronchiectasis in this patient.

Carcinoid tumorlet is a nodular proliferation of neuroendocrine cells and arises from focal areas of bronchial and bronchiolar Kultschitsky cells, usually associated with diffuse bronchiectasis and intralobar sequestration (7–10). It is not larger than 5 mm (2). D’Agati and Perzin found that pulmonary tumor-lets had tumor characteristics since they could metastasize to the lymph nodes (11). Chromogranin A, CD56 and synaptophysin are the most useful neuroendocrine immunohistochemical markers (12), and low levels of Ki-67 can help to distinguish neuroendocrine from small-cell carcinoma. In the present case the nodule was 0.4 cm, and positive for synaptophysin, chromogranin A, CK-L, Ki-67 (<1% positive), TIF1 and CD56, and negative for S-100, so carcinoid tumorlet was diagnosed. Certain studies claim that carcinoid tumorlet may be induced by hypoxia caused by bronchiectasis or other chronic bronchitis lesions (13). In the case presented here, both bronchiectasis and carcinoid tumorlet in PS were found in the left lower lung field.

Hocking *et al* found that the risk of developing carcinoid tumors with breast cancer is more than double the expected rate (14); however, the reasons for this are not understood. In the present case, the patient was diagnosed with breast cancer before the appearance of the carcinoid tumorlet.

In the case presented here, the patient suffered PS and bronchiectasis as well as carcinoid tumorlet in PS following the diagnosis of breast cancer three years earlier. It is possible that both breast cancer and bronchiectasis are the result of a carcinoid tumorlet, and that PS may be acquired and secondary to occluding carcinoid tumorlet.
